# A Simple and Reliable Method for the Determination of Isorhapontigenin in Murine Biological Matrices: Application in a Tissue Distribution Study

**DOI:** 10.3390/molecules30173635

**Published:** 2025-09-05

**Authors:** Yuhui Yang, Hongrui Jin, Boyu Liao, Feifei Gao, Yihan Yang, Xinyi Wang, Zhang Liu, Jingsi Liang, Jingbo Wang, Paul Chi-Lui Ho, Hui Liu, Hai-Shu Lin

**Affiliations:** 1 School of Pharmacy, Shenzhen University Medical School, Shenzhen University, Shenzhen 518055, China; 2 College of Pharmacy, Shenzhen Technology University, Shenzhen 518118, China; 3 School of Pharmacy, Monash University Malaysia, Jalan Lagoon Selatan, Bandar Sunway, Subang Jaya 47500, Malaysia; 4 Quality and Standards Academy, Shenzhen Technology University, Shenzhen 518118, China

**Keywords:** isorhapontigenin, resveratrol, nutraceutical, bioavailability, biodistribution

## Abstract

Isorhapontigenin (*trans*-3,5,4′-trihydroxy-3′-methoxystilbene; ISO), a dietary derivative of resveratrol (*trans*-3,5,4′-trihydroxystilbene; RES), exhibits diverse health-promoting properties. To facilitate its potential development as a nutraceutical, a simple and reliable high-performance liquid chromatography (HPLC) method was developed and validated for the quantification of ISO in various murine biological matrices. Chromatographic separation was achieved with a reversed-phase HPLC column through a 17 min gradient delivery of a mixture of acetonitrile and formic acid (0.1% *v*/*v*) at a flow rate of 1.5 mL/min at 50 °C. Quantification was performed using ultraviolet (UV) detection at 325 nm, with a lower limit of quantification (LLOQ) of 15 ng/mL in both plasma and tissue homogenate samples. The method demonstrated excellent selectivity, accuracy, and precision, and ISO remained stable under the tested conditions. This method was subsequently employed to investigate the tissue distribution of ISO in mice following oral administration at a dose of 200 µmol/kg (equivalent to 51.7 mg/kg). ISO was rapidly absorbed and extensively distributed across major pharmacologically relevant organs. Despite its limited aqueous solubility, its oral absorption was not significantly compromised. Given its oral bioavailability and broad tissue distribution, ISO represents a promising candidate for further nutraceutical development.

## 1. Introduction

**Figure 1:**
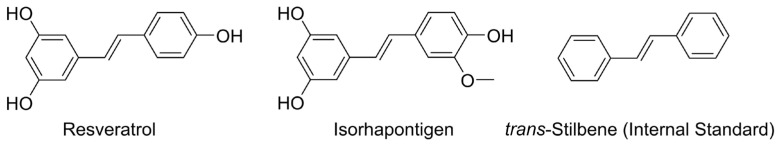
Chemical structure of resveratrol, isorhapontigenin and *trans*-stilbene (internal standard).

Resveratrol (*trans*-3,5,4′-trihydroxystilbene; RES; Figure 1), one of the most well-known nutraceuticals, occurs naturally in dietary sources such as grapevine, red wine, cranberry, blueberry, bilberry, peanut, and various herbs [1,2,3]. Since the turn of the millennium, RES has attracted considerable attention in the biomedical research community due to its reported potential benefits in managing a wide range of medical conditions, including Alzheimer’s disease, cancer, cardiovascular diseases, chronic kidney disease, diabetes, nonalcoholic fatty liver disease, obesity, and metabolic syndrome. Increasing interest is also being directed toward its naturally occurring dietary derivatives [4].

Isorhapontigenin (*trans*-3,5,4′-trihydroxy-3′-methoxystilbene; ISO; Figure 1), a methoxylated RES analog is present in grapes and a variety of medicinal plants [5,6,7,8,9]. ISO, akin to RES, displays nutraceutical potentials and its anti-aging [10], anti-cancer [11], anti-diabetic [12], anti-inflammation [6,7], anti-oxidation [9], cardio- [13], hepato- [6], neuro- [14] and renal [15] protective activities have been reported in pre-clinical studies. In our previous study [16], we evaluated the therapeutic potential of ISO in primary airway epithelial cells isolated from patients with chronic obstructive pulmonary disease (COPD), using RES as a comparator. ISO significantly suppressed key inflammatory pathways that drive COPD pathogenesis and demonstrated stronger anti-COPD activity than RES. Notably, its anti-inflammatory effects were mediated through a unique mechanism independent of corticosteroid signaling, suggesting potential utility in treating corticosteroid-resistant inflammation in COPD. Given the global burden of COPD affecting hundreds of millions worldwide, it is of great scientific interest to further explore the nutraceutical potential of ISO. In our recent study, the impacts of ISO on metabolism and health were assessed in Sprague Dawley rats after two weeks of daily oral administration at a dose of 100 µmol/kg/day [17]. The rats receiving ISO treatment showed less body weight gain and displayed healthier blood cholesterol levels, while no significant toxicity was observed. Metabolomic examination of plasma, hepatic, cardiac, and brain samples indicated an anti-diabetic, cardio-, hepato-, and neuro-protective role of ISO. Interestingly, although RES and ISO share similar chemical structures, their metabolic impacts appear distinct.

Oral bioavailability, which determines how efficiently an active compound is absorbed and reaches systemic circulation after oral administration, is one of the critical determinants in the successful development of nutraceuticals. Upon oral dosing, ISO demonstrated rapid absorption in both mice and rats [16,18,19]. We further confirmed that aqueous solubility was not a limiting factor for its oral absorption, while its absolute oral bioavailability was approximately 20–30% in rats [16,19]. Moreover, one-week repeated dosing did not result in any significant changes in its oral bioavailability [16,19]. Taken together, these results highlight ISO as a strong candidate for continued nutraceutical development.

However, plasma levels alone are insufficient to optimize the medicinal application of ISO, as its distribution in major target organs such as the brain, lung, and heart remains largely unknown. A comprehensive understanding of tissue-specific exposure is essential, since pharmacological effects are often determined by local concentrations rather than systemic levels. Ideally, confirming its bioavailability in individual organs and tissues would provide critical insights into how ISO exerts its biological effects, thereby guiding the identification of suitable nutraceutical indications across a range of medical conditions.

Sensitive bioanalytical methods play an irreplaceable role in pharmacokinetic studies. In our previous work, plasma profiles of ISO in rats were analyzed using liquid chromatography–tandem mass spectrometry (LC-MS/MS) [16,19,20]. This method demonstrated excellent sensitivity, with a lower limit of quantification (LLOQ) of 1 ng/mL in rat plasma. However, despite employing a straightforward protein-precipitation sample clean-up strategy, this LC-MS/MS method failed to quantify ISO in other biological matrices—particularly liver and lung homogenates—due to pronounced matrix effects (unpublished data). Tissue homogenates from solid organs such as intestines, liver, kidney, and lung are inherently more complex and contain abundant endogenous compounds, which can introduce interference and markedly impair ionization efficiency. It is widely recognized that protein-precipitation alone is insufficient for LC-MS/MS analysis of such complex matrices.

High-performance liquid chromatography (HPLC) with ultraviolet (UV) detection is still widely used in bioanalysis when sensitivity is not a critical concern, owing to its relative insensitivity to matrix effects. Compared with more sophisticated techniques, it is also more cost-effective and accessible. Therefore, to ensure both reliability and affordability, we developed and validated a simple and reliable HPLC method for quantifying ISO in various murine biological matrices in the present study. The biodistribution of ISO was then profiled in mice following oral administration. To our knowledge, this is the first report detailing the tissue distribution of ISO. These findings provide critical insights for optimizing its nutraceutical application.

## 2. Results

### 2.1. Assay Development

The chromatographic conditions—including mobile phase composition and gradient program—were optimized to produce sharp, symmetrical peaks for ISO. As a polyphenol, ISO’s retention on the C18 stationary phase is strongly influenced by pH (unpublished data). To maintain assay simplicity, ISO was separated from its primary metabolite, IS and other matrix components using a 17 min gradient elution of acetonitrile and 0.1% formic acid (*v*/*v*). Although increasing the injection volume is a common approach to enhance the sensitivity of assays targeting stilbenes, we found that volumes exceeding 10 μL led to peak distortion and asymmetry. Therefore, the injection volume was fixed at 10 μL.

### 2.2. Assay Validation

This HPLC-UV method was validated by the examination of its selectivity, sensitivity, precision, accuracy, matrix effect, and stability profiles of ISO in accordance with EMA and US FDA guidelines [21,22].

**Figure 2:**
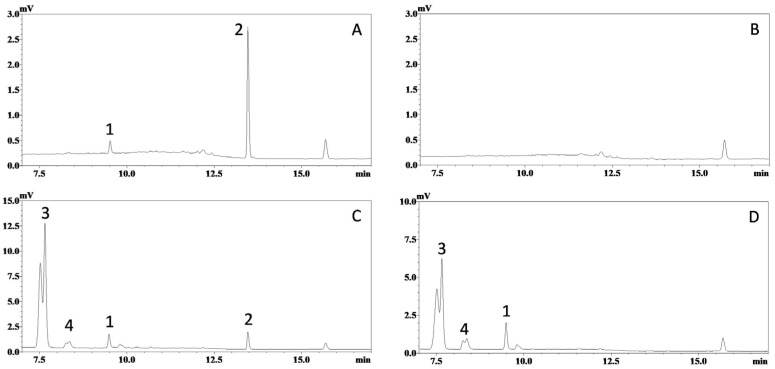
Representative chromatograms of mouse plasma samples. Ultraviolet absorbance at 325 nm was recorded for: (**A**) blank mouse plasma spiked with ISO (corresponding to 75 ng/mL in plasma) and *trans*-stilbene (internal standard, corresponding to 1200 ng/mL in plasma); (**B**) blank mouse plasma; (**C**) plasma sample collected 20 min after oral administration of ISO (200 µmol/kg or 51.7 mg/kg) with internal standard; and (**D**) plasma sample collected 20 min after oral administration of ISO (200 µmol/kg) without internal standard. Peaks: 1, ISO; 2, internal standard; 3 & 4, unidentified metabolites.

The selectivity of this HPLC-UV assay was confirmed, as no significant interference was observed at the retention times of ISO (~9.5 min, peak 1) and IS (~13.4 min, peak 2) in the chromatograms (λ = 325 nm) from blank mouse plasma and tissue homogenate samples from at least six mice. Representative UV chromatograms of a mouse plasma sample spiked with ISO (75 ng/mL) and IS (1200 ng/mL), and a blank plasma sample, are shown in Figure 2A,B, respectively. Representative chromatograms of plasma samples collected after oral dosing of ISO are shown in Figure 2C (with IS), D (without IS).

**Figure 3:**
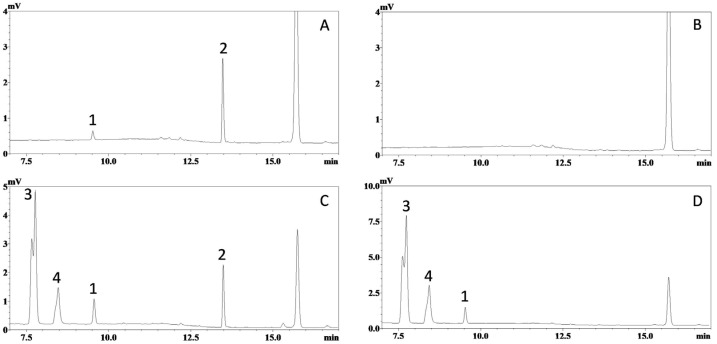
Representative chromatograms of mouse hepatic homogenate samples. Ultraviolet absorbance at 325 nm was recorded for: (**A**) blank mouse hepatic homogenate spiked with ISO (corresponding to 75 ng/mL in homogenate and 450 ng/mL in tissue) and *trans*-stilbene (internal standard, corresponding to 1200 ng/mL in homogenate); (**B**) blank mouse hepatic homogenate; (**C**) hepatic sample collected 20 min after oral administration of ISO (200 µmol/kg or 51.7 mg/kg) with internal standard; and (**D**) hepatic sample collected 20 min after oral administration of ISO (200 µmol/kg) without internal standard. Peaks: 1, ISO; 2, internal standard; 3 and 4, unidentified metabolites.

It is generally believed that the bioanalysis of tissue samples is more challenging than that of plasma samples, as tissue matrices are more complex, have higher levels of endogenous compounds, and often require more labor-intensive homogenization and extraction procedures. Fortunately, the selectivity of this assay was successfully validated in the homogenates of different tissues. Representative chromatograms obtained from hepatic tissue, one of the most challenging tissues for bioanalysis, are displayed in Figure 3, while chromatograms from other tissues are listed in Supplementary Materials (Figure S1–S9). Again, no significant interference for ISO and IS was observed. This clearly demonstrates the selectivity of the HPLC-UV method.

The LLOQ of ISO using this HPLC-UV method was determined to be 15 ng/mL in plasma. At this concentration, the signal-to-noise ratio exceeded 5:1 (chromatogram not shown). Both intra-day and inter-day analyses demonstrated mean accuracy within 100 ± 10%, with RSDs not exceeding 10% (Table 1). The LLOQs of ISO in various tissue homogenates were also found to be 15 ng/mL (corresponding to 90 ng/g in tissue). Similarly, in hepatic homogenate, intra-day and inter-day analyses showed mean accuracy within 100 ± 10%, with RSDs not exceeding 10% (Table 1). In other homogenates, intraday analyses also yielded mean accuracy within 100 ± 15%, with RSDs not exceeding 15% (Supplementary Table S1). Compared with the LC-MS/MS approach, the HPLC-UV method exhibited a higher LLOQ but enabled quantification of ISO in various tissues.

The calibration curves were constructed using chromatographic data obtained from different biological matrices, covering a concentration range of 15–2000 ng/mL. All calibration curves exhibited excellent linearity, with correlation coefficients (*R*^2^) greater than 0.99 (Table 2).

The accuracy and precision of the HPLC-UV assay were evaluated using QC samples (15, 45, 175, 800, and 1600 ng/mL) prepared in various biological matrices. Intraday and inter-day analyses were conducted in plasma and liver homogenate (Table 1), while only inter-day analyses were performed for homogenates of other tissues (Supplementary Materials, Table S1). The assay demonstrated acceptable accuracy and precision, with mean accuracies within 100 ± 15% and RSDs not exceeding 15% across all QC levels.

The absolute recovery (%) and matrix effect were assessed using plasma and hepatic homogenate as representative matrices. The extraction efficiency was high, with mean absolute recoveries within 100 ± 15% and relative standard deviations (RSDs) not exceeding 10% across all QC levels (Table 3). Similarly, the matrix effect was negligible, as the IS-normalized matrix factors remained within 1.00 ± 0.15, with RSDs below 5% (Table 3).

The stability profiles of ISO and IS stock solutions under our handling conditions were examined and confirmed (Table 4).

In bioanalysis, analyte stability in biological matrices is crucial for ensuring accurate and reliable quantification. Instability can result in degradation or transformation during sample collection, storage, or processing, potentially compromising data integrity and study outcomes. Therefore, the stability of ISO in plasma was thoroughly evaluated under various storage conditions (Table 5). Assessing ISO stability in intact tissues is challenging, as replicating the native tissue environment is difficult. Accordingly, only short-term stability (6 h on ice) was assessed in tissue homogenates (Table 6). For post-preparative stability, hepatic homogenate—the most complex matrix—was selected as a representative. ISO exhibited good stability under all tested conditions.

As demonstrated, a simple and reliable HPLC-UV method was successfully developed and validated for the quantification of ISO in various mouse biological matrices, enabling comprehensive biodistribution studies of ISO in mice.

### 2.3. Application to Biodistribution Stduy

**Figure 4:**
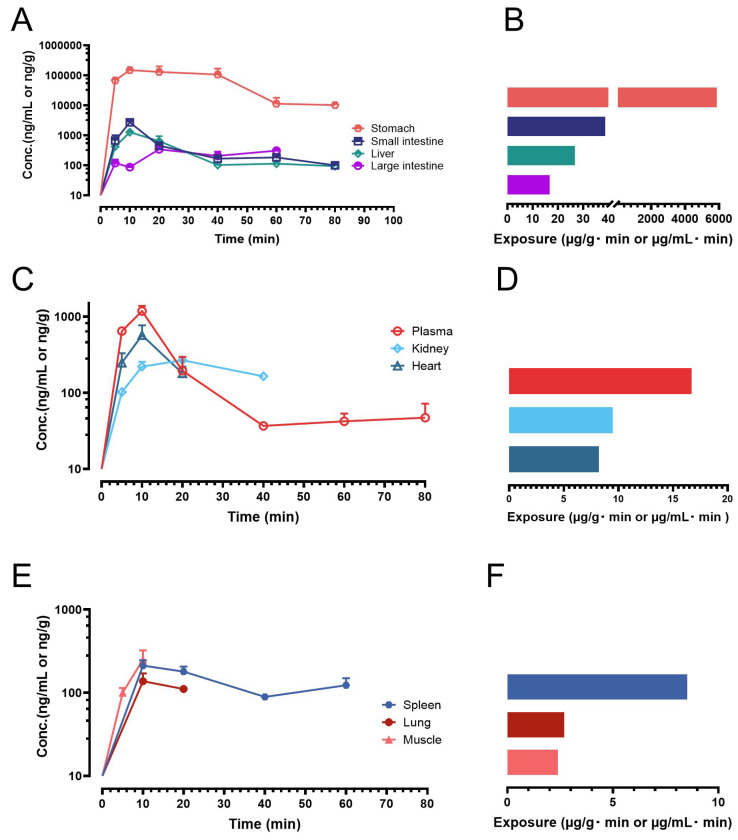
Biodistribution profiles and exposures of ISO in mice following oral administration (200 µmol/kg or 51.7 mg/kg). Symbol represents mean value while error bar represents SD. (**A**) Concentration–time profiles of ISO in the stomach, small intestine, liver, and large intestine; (**B**) Exposure data (AUC) for the stomach, small intestine, liver, and large intestine; (**C**) Concentration–time profiles of ISO in the plasma, kidney and heart; (**D**) Exposure data for the plasma, kidney and heart; (**E**) Concentration–time profiles of ISO in the spleen, lung and muscle; (**F**) Exposure data for the spleen, lung and muscle.

Following oral administration of ISO at a dose of 200 µmol/kg (equivalent to 51.7 mg/kg), three to four mice (*n* = 3 or 4) were euthanized at each predetermined time point (5, 10, 20, 40, 60, and 80 min), and biological samples were collected. ISO concentrations were quantified using our validated HPLC-UV method. Exposure levels (AUC) in plasma and other individual biological matrices were calculated based on the mean concentrations at each time point. The resulting concentration–time profiles and exposure data of ISO are presented in Figure 4.

Previous studies have demonstrated that ISO undergoes rapid oral absorption in both mice and rats, with peak plasma concentrations occurring within 15 min post-dosing [16,18,19]. These findings were confirmed in the present study, where ISO was rapidly absorbed, with mean concentrations in plasma and multiple tissues peaking at 10 min (Figure 4). However, ISO was also eliminated quickly. In plasma, ISO became unquantifiable in one out of four mice by 40 min. Similarly, its concentrations in the kidney, liver, and heart became undetectable in some mice as early as 20 min post-dosing. ISO was detectable in the lung and muscle mainly between 10 and 20 min. Notably, ISO exhibited particularly high exposure in the gastrointestinal tract, including the stomach, small intestine, and large intestine. The exposure ranking was as follows: stomach > small intestine > liver > large intestine > plasma > kidney > heart > spleen > lung > muscle. Interestingly, ISO was also able to penetrate lipophilic tissues such as the brain and fat. While ISO was detectable in many brain and fat samples, only a few reached quantifiable levels (≥75 ng/mL). These results clearly indicate that, following a single oral dose, ISO was systemically available in plasma as well as in many major drug-targeting organs.

## 3. Discussion

In our previous study, the metabolism of ISO was investigated using an enzymatic-hydrolysis approach, which revealed extensive glucuronidation [20]. Although ISO sulfation was not detected, piceatannol exhibited extensive sulfation under the same conditions [20]. Likewise, phase II metabolism of RES—including both glucuronidation and sulfation—has been established as its primary metabolic pathway. Both conjugation reactions increase polarity, so these metabolites should elute earlier than ISO in reversed-phase chromatography. Consistent with this, peaks 3 and 4 (Figure 2 and Figure 3)—attributed to ISO metabolites—were observed in plasma/tissue homogenate chromatograms following ISO administration. These peaks likely represent coelution of multiple conjugates, since ISO’s several hydroxyl groups can be modified at different positions. However, as our aim was to develop and validate a straightforward HPLC-UV method, metabolite quantification via enzymatic hydrolysis was not performed in the present study.

The simplicity of this HPLC-UV method remains its primary advantage. Plasma and various tissue homogenates can be analyzed with satisfactory selectivity and minimal matrix effect, requiring only a direct protein-precipitation sample procedure for sample preparation. With a LLOQ of 15 ng/mL in both plasma and tissue homogenates, this method enabled the successful profiling of ISO bio-distribution in mice. Notably, ISO exhibits negligible biological activity at concentrations near this threshold, demonstrating that this straightforward HPLC-UV method is well-suited for assessing its biodistribution. However, in addition to the relatively low sensitivity, the long assay time (17 min) represents another major limitation of this method. Stilbene compounds generally exhibit strong UV absorbance, and based on published experience, similar sample preparation procedures and HPLC detection conditions typically achieve an LLOQ of 10–15 ng/mL in plasma samples [23,24]. Collectively, this straightforward approach may also be applicable to future biodistribution studies involving dietary or herbal stilbenes.

Although LC-MS/MS has established a lower limit of quantitation (LLOQ) as low as 1 ng/mL in plasma matrices [16,19,20], its translation to biodistribution studies involving complex tissue homogenates remains technically challenging. Exhaustive sample preparation workflows—encompassing liquid–liquid extraction (LLE) and/or solid-phase extraction (SPE)—are indispensable for mitigating matrix effects, thereby imposing inherent constraints on the method’s scalability. The cumulative error propagation, protracted analytical timelines, and prohibitive costs associated with these multistep protocols compromise the reproducibility and operational efficiency of LC-MS/MS for high-throughput biodistribution profiling. In contrast, the HPLC-UV method presents a viable alternative when sensitivity requirements are modest.

For ISO, glucuronidation and/or sulfation act as a double-edged sword. On the one hand, these phase II modifications yield conjugates that are more hydrophilic, display diminished membrane permeability, and consequently may exhibit reduced biological activity. Additionally, these conjugates are typically eliminated more efficiently via renal excretion. On the other hand, glucuronide and sulfate metabolites are chemically labile and can be enzymatically cleaved by glucuronidases and sulfatases, regenerating the parent aglycone. This reversible biotransformation illustrates the intricate balance between conjugation and deconjugation, emphasizing the complexity of phase II metabolic processes and their significant roles in modulating the pharmacokinetic and pharmacodynamic profiles of active compounds.

Although plasma pharmacokinetic studies of ISO have been conducted in both mice and rats [18,19,20], this is the first study to examine its biodistribution. We confirmed that ISO is bioavailable in various drug-targeting organs. Notably, ISO was particularly abundant in the gastrointestinal tract, including the stomach, small intestine, and large intestine. Although it is widely recognized that the small intestine is the primary site of nutrient and drug absorption following oral administration, the presence of ISO in the stomach may partly reflect unabsorbed compound. However, the relatively high plasma concentrations observed as early as 5 min post-dosing strongly suggest the likelihood of gastric absorption. As a polyphenolic compound, ISO may exhibit enhanced membrane permeability in the stomach due to reduced ionization under acidic conditions. Notably, our findings indicate a potential added value of ISO as a nutraceutical for gastric health applications. Following absorption, ISO was rapidly distributed via the bloodstream, resulting in substantial exposure in highly perfused organs such as the liver, heart, and kidneys. Of note, several ISO derivatives—including resveratrol, oxyresveratrol, and piceatannol—also exhibit rapid oral absorption [20,25], suggesting they may undergo gastric absorption as well. Exploring this hypothesis in future studies would hold scientific merit.

Although ISO has demonstrated neuroprotective potential in rats following repeated intraperitoneal dosing [14], its limited brain penetration after oral administration raises concerns about its suitability as a nutraceutical for neurodegenerative diseases that require sustained therapeutic levels through chronic oral use. In contrast, pterostilbene—a partially methoxylated derivative of RES—shows significantly improved brain bioavailability and appears to be a more promising candidate for the treatment of such conditions [26].

In addition to its substantial accumulation in highly perfused organs such as the liver, heart, and kidneys, ISO also demonstrated notable distribution to the spleen, suggesting a potential involvement of the lymphatic system in its absorption. If ISO is indeed absorbed via the lymphatic pathway, its nutraceutical applications could be expanded to target conditions related to the lymphatic or immune systems, including inflammation, immune modulation, and lymphatic disorders. This represents a novel observation highlighted in the current study.

It is widely recognized that many substrates undergo phase II metabolism more efficiently in mice, followed by rats, with humans generally exhibiting the lowest metabolic activity in terms of both rate and extent. Compared to rats [16,19], plasma ISO concentrations decline more rapidly in mice following oral administration with a similar formulation, suggesting more efficient metabolism in mice. Although the metabolic and pharmacokinetic profiles of ISO in humans remain unclear, a more favorable profile may be expected, supporting its potential application as a nutraceutical for health-promoting purposes.

Despite being administered as a suspension without any solubility-enhancing excipients, ISO was rapidly absorbed, with mean concentrations in plasma and various tissues peaking at 10 min (Figure 4). These findings suggest that poor solubility is not a major barrier to ISO’s oral bioavailability; therefore, formulation strategies aimed at enhancing solubility or dissolution are unlikely to substantially improve its nutraceutical effectiveness. In contrast, due to ISO’s relatively rapid systemic elimination, a sustained-release formulation may offer a more effective strategy for nutraceutical use, as it could extend systemic exposure, maintain therapeutic concentrations over time, and potentially enhance biological activity in target tissues. Numerous cost-effective sustained-release oral dosage forms have been developed and implemented in clinical settings. Among these, matrix tablets, osmotic pump tablets, and reservoir systems represent feasible approaches for the sustained-release formulation of ISO, potentially offering particular advantages for its nutraceutical use in gastrointestinal tract-related conditions.

Over the past two decades, we have extensively investigated the impact of aqueous solubility on the oral bioavailability of resveratrol (RES) and its naturally occurring and synthetic derivatives. Based on these studies, we propose the following empirical guideline: for stilbenes containing three or more hydroxyl groups—such as RES, oxyresveratrol, piceatannol, and ISO—aqueous solubility does not pose a significant barrier to oral absorption. In contrast, for methoxylated stilbenes or those with fewer than three hydroxyl groups, the use of solubility-enhancing excipients or strategies is necessary to facilitate effective oral delivery. This rule of thumb may serve as a practical tool for selecting suitable RES derivatives for nutraceutical development.

## 4. Materials and Methods

### 4.1. Special Precautions

All laboratory procedures involving the manipulations of stilbenes were carried out in a dimly lit environment to prevent their photo-isomerization [17].

### 4.2. Chemicals and Reagents

Isorhapontigenin (*trans*-3,5,4′-trihydroxy-3′-methoxystilbene, ISO, Figure 1, purity: 96.0%), *trans*-stilbene (internal standard, Figure 1, purity: 98.0%) and L-ascorbic acid (vitamin C; purity: 99.0%) were purchased from Tokyo Chemical Industry (Tokyo, Japan). All other chemicals and reagents were of at least reagent grade. Ultra-pure water (18.2 MΩ·cm at 25 °C) and chromatographic grade acetonitrile and methanol were used throughout the study. Murine blank plasma was purchased from Sbjbio (Nanjing, China).

### 4.3. HPLC

All HPLC analyses were carried out using a Shimadzu (Kyoto, Japan) LC-20AT Liquid Chromatography system. This HPLC system consisted of an LC-20AT Solvent Delivery Unit Block, a SIL-20AXR Autosampler, a CTO-20A Column Oven, an SPD-20A UV-VIS Detector, and a CBM-20A Lite System Controller. The HPLC was operated via the LabSolutions Single LC-PDA version 1.25 software workstation, and chromatographic data analysis was also carried out using the same software.

A RP-HPLC column (Agilent (Santa Clara, CA, USA) ZORBAX Eclipse Plus C18: 250 × 4.6 mm i.d., 5 µm), protected by a guard column (Agilent ZORBAX Eclipse Plus C18: 12.5 × 4.6 mm i.d., 5 µm) was used to quantify ISO in various murine biological matrices. Chromatographic separation was obtained through a 17 min gradient delivery of a mixture of acetonitrile and formic acid (0.1% *v*/*v*) at a flow rate of 1.5 mL/min at 50 °C. The gradient schedule was: (a) 0–2 min, acetonitrile: 15%; (b) 2–6 min, acetonitrile: 15–40%; (c) 6–9 min, acetonitrile: 40–90%; (d) 9–14 min, acetonitrile: 90%; (e) 14–17 min, acetonitrile: 15%. Ultraviolet (UV) absorbance at 325 nm (maximal UV absorption wavelength of ISO) was recorded.

### 4.4. Sample Preparation

Stock solutions of ISO and *trans*-stilbene (internal standard, IS) were prepared in DMSO at a final concentration of 1 mg/mL. These solutions were stored at −20 °C and protected from light. The calibration standard and quality control (QC) samples were prepared by serial dilution of the ISO stock solution with pooled blank murine plasma or respective tissue homogenate.

For the cleanup of plasma samples, three volumes of acetonitrile (containing IS at 400 ng/mL) were added to one volume of murine plasma to precipitate proteins [23,24]. After vortexing for 20 s, the samples were centrifuged at 1503× *g* for 10 min at 4 °C. The supernatant was carefully transferred to a glass insert preloaded in an autosampler vial. During each HPLC analysis, 10 μL of supernatant was injected into the HPLC system. The minimum plasma volume required per analysis was 25 μL.

For tissue samples, namely brain, fat, heart, small intestine, large intestine, kidney, liver, lung, muscle, spleen, and stomach, approximately 35 mg of each tissue was weighed and finely minced with scissors. The samples were homogenized in a 0.2% ascorbic acid solution (freshly prepared daily) in 50% methanol (*v*/*v*) at a ratio of 1:5 (*w*/*v*) for up to three cycles using a high-throughput tissue homogenizer (Wonbio-L, Wonbio, Shanghai, China) operating at 60 Hz for 60 s per cycle. Subsequently, three volumes of acetonitrile (containing IS at 400 ng/mL) were added to one volume of tissue homogenate. Following vigorous vortexing, the samples were centrifuged at 1902× *g* for 10 min at 15 °C. The clear supernatant was carefully transferred into an HPLC vial.

During each HPLC analysis, 10 μL of supernatant was injected into the HPLC system.

### 4.5. Assay Validation

This HPLC method was validated in accordance with EMA and US FDA guidelines [21,22], with comprehensive evaluation of selectivity, sensitivity, linearity, accuracy (analytical recovery), intra- and inter-day precision, absolute recovery, matrix effect and stability.

The selectivity of this assay was assessed by comparing the chromatograms of blank matrices from six individual mice with those of the same samples spiked with ISO and *trans*-stilbene.

The sensitivity of this assay was represented by the lower limit of quantification (LLOQ), which was defined as the minimal concentration that produces a signal-to-noise ratio of no less than 5, with acceptable accuracy (mean analytical recovery: 80–120%) and precision (relative standard deviation (RSD) ≤ 20%).

The peak area ratio between ISO and the internal standard was used as the analytical response. Calibration curves were constructed using GraphPad Prism 8 (La Jolla, CA, USA), where *x* represented the concentration of ISO, y represented the analytical response, and *1*/*x*^2^ was used as the weighting factor, as routinely applied [23,24]. Calibration standards at concentrations of 15, 30, 75, 200, 500, 1200, 1800, and 2000 ng/mL were used to construct the calibration curve and assess linearity in all biological matrices. During quantification of actual samples from the biodistribution study, those with ISO concentrations exceeding 2000 ng/mL were appropriately diluted with blank matrix to bring them within the calibration range.

Intra- and inter-day accuracy and precision were evaluated using QC samples at five different concentrations: 15, 45, 175, 800, and 1600 ng/mL. Accuracy was assessed by expressing the measured concentrations as percentages of the corresponding nominal concentrations. Precision was determined by calculating the relative standard deviation (RSD). The assay was considered acceptable if the mean analytical recovery was within 80–120% and the RSD was ≤ 20% at LLOQ. For all other concentrations, the mean analytical recovery had to be within 85–115%, with an RSD ≤ 15% [21,22].

The absolute recovery (%) and matrix effect were evaluated using plasma and hepatic homogenate as representative matrices. Absolute recovery was calculated by comparing the ISO peak area in blank matrix samples spiked with ISO to that in neat solutions containing the same concentration of ISO. The matrix effect was assessed using individual blank matrix samples obtained from six different mice. The matrix factor was defined as the ratio of the ISO or IS peak area in matrix samples (where ISO and the IS were spiked into the supernatant obtained after protein precipitation) to that in neat solutions spiked with the same concentrations. The IS–normalized matrix factor was also calculated. A matrix effect was considered negligible if the RSD of the IS–normalized matrix factor was less than 15%.

The stability of ISO was evaluated under various storage conditions. Stock solution stability was assessed after storage at room temperature (25 °C) and –20 °C. The stability of ISO in plasma was examined under the following conditions: short-term storage (25 °C for 24 h), long-term storage (–80 °C for 52 days), three freeze–thaw cycles, and post-preparative stability (samples kept in an autosampler at 4 °C for more than 24 h). As it is difficult to evaluate ISO stability directly in tissues, only the short-term stability in tissue homogenates was assessed (6 h on ice). Similarly, post-preparative stability in processed tissue samples was assessed using liver homogenate as a representative, due to the complexity of the hepatic matrix. Stability was considered acceptable if 85–115% of the initial ISO concentration remained.

### 4.6. Animals

The study was conducted in accordance with the ARRIVE guidelines [27]. The study design and animal handling protocol reviewed and approved by the Ethics Committee of the Shenzhen Technology University (20240803) on 03 August 2024.

Specific pathogen-free C57BL/6 mice (male; 6–8 weeks old; weight: 18–21 g) were purchased through Shenzhen Glorybay Biotech Co. (Shenzhen, China). The animals had *ad libitum* access to food and water prior to dosing. ISO was administered as an aqueous suspension (vehicle: 0.3% sodium carboxymethylcellulose with 0.2% L-ascorbic acid; ISO concentration: 5 mg/mL) via oral gavage at a dose of 200 µmol/kg (equivalent to 51.7 mg/kg, dosing volume: 10.34 mL/kg). At each predetermined time point (5, 10, 20, 40, 60 and 80 min) three to four mice (*n* = 3 or 4) were euthanized through cervical dislocation. Blood samples were collected into heparinized centrifuge tubes and centrifuged to obtain plasma. Following blood collection, the mice were dissected, and major organs/tissues—including the brain, abdominal fat, heart, small intestine, large intestine, kidney, liver, lung, abdominal muscle, spleen, and stomach—were harvested. The organs and tissues were cut into small pieces and rinsed with isotonic saline before being blotted dry. All collected biological samples were placed on dry ice. At the end of the in vivo study, all samples were stored at –80 °C in a deep freezer until HPLC analysis.

### 4.7. Pharmacokinetic Analysis

Non-compartmental pharmacokinetic analysis was performed using WinNonlin Standard Version 1.0 (Scientific Consulting Inc., Apex, NC, USA). Plasma and tissue exposure, expressed as the area under the concentration–time curve (AUC), was calculated using the mean values of the individual mice [26].

### 4.8. Statistics

Data are commonly presented as mean ± standard deviation (SD).

## 5. Conclusions

In this study, a simple and reliable HPLC-UV method was successfully developed and validated for the quantification of ISO in various murine biological matrices. This bioanalytical method was subsequently applied to investigate the biodistribution of ISO in mice. ISO was rapidly absorbed following oral administration and widely distributed across major drug-targeting organs. Given its oral bioavailability and broad tissue distribution, ISO presents as a promising nutraceutical candidate for further development.

## Figures and Tables

**Table 1:** Accuracy and precision of isorhapontigenin in mouse plasma and hepatic homogenate *.

Type of Matrice	Nominal Concentration in Matrice (ng/mL)	Intra-Day			Inter-Day		
		Measured Concentration (ng/mL)(Mean ± SD)	Precision (RSD%)	Mean Accuracy (%)	Measured Concentration (ng/mL) (Mean ± SD)	Precision (RSD%)	Mean Accuracy (%)
Plasma	15.0	15.4 ± 1.0	6.4	102.5	16.1 ± 1.0	6.2	107.1
	45.0	44.7 ± 0.7	1.6	99.4	45.2 ± 1.1	2.4	100.5
	175	172.7 ± 3.6	2.1	98.6	176.4 ± 4.6	2.6	100.9
	800	792.5 ± 14.8	1.9	99.1	801.4 ± 14.0	1.7	100.2
	1600	1612.3 ± 16.3	1.0	100.8	1625.8 ± 30.1	1.9	101.6
Liver	15.0	14.9 ± 1.2	8.0	99.6	116.1 ± 1.5	9.0	107.0
	45.0	45.5 ± 1.0	2.2	101.2	47.4 ± 1.9	3.9	105.4
	175	170.7 ± 6.3	3.7	97.5	177.2 ± 11.1	6.2	101.2
	800	762.2 ± 10.7	1.4	95.3	780.1 ± 39.2	5.0	97.5
	1600	1519.5 ± 24.0	1.6	95.0	1549.7 ± 36.7	2.4	96.9

* Intraday analyses were conducted in six replicates, whereas inter-day analyses were performed over three consecutive days, with six replicates per day.

**Table 2:** Calibration curves of ISO in different matrices.

Biological Matrix	Calibration Equation	*R* ^2^
Plasma	*y = 0.6659x − 0.0045*	0.9993
Liver	*y = 0.6653x + 0.0013*	0.9984
Heart	*y = 0.6032x + 0.0002*	0.9979
Kidney	*y = 0.6092x − 0.0035*	0.9980
Brain	*y = 0.5739x − 0.0040*	0.9992
Spleen	*y = 0.6311x − 0.0004*	0.9992
Stomach	*y = 0.6613x + 0.0107*	0.9970
Lung	*y = 0.6508x − 0.0018*	0.9981
Fat	*y = 0.7034x − 0.0024*	0.9977
Small Intestine	*y = 0.6191x − 0.0018*	0.9987
Large Intestine	*y = 0.6350x − 0.0008*	0.9979
Muscle	*y = 0.6213x − 0.0045*	0.9980

**Table 3:** Absolute recovery (%) and internal standard normalized matrix factor *.

Matrice	Concentration (ng/mL)	Absolute Recovery (%)		Matrix Factor	
		Mean ± SD	RSD	Mean ± SD	RSD
Plasma	45.0	101.4 ± 5.5	5.5	0.9 ± 3.5	3.6
	800	100.1 ± 1.3	1.3	1.0 ± 1.5	1.4
	1600	100.3 ± 1.6	1.6	1.0 ± 0.9	0.9
Hepatic Homogenate	45.0	94.5 ± 3.7	3.9	1.1 ± 0.0	2.5
	800	94.9 ± 1.4	1.4	1.1 ± 0.0	2.3
	1600	89.5 ± 1.9	2.2	1.1 ± 0.0	0.0

* *n* = 6.

**Table 4:** Stability of stock solutions *.

Storage Condition	Concentration (mg/mL)	Remaining (%, Mean ± SD)
Isorhapontigenin stored at room temperature for 24 h	1.00	94.6 ± 0.7
Isorhapontigenin stored at −20 °C for 20 days	1.00	104.7 ± 1.4
Internal standard stored at room temperature for 24 h	0.40	94.7 ± 0.3
Internal standard stored at −20 °C for 20 days	0.40	105.3 ± 0.7

* *n* = 6.

**Table 5:** Stability of plasma samples *.

Stability Profiles	Remaining (%, Mean ± SD)	
	45.0 ng/mL	1600 ng/mL
After three freeze and thaw cycles	100.6 ± 4.4	104.8 ± 1.1
Short-term stability (24 h at room temperature)	102.0 ± 3.7	101.74 ± 0.9
Post-preparative stability (samples kept in an autosampler at 4 °C for at least 24 h)	102.4 ± 2.7	101.65 ± 1.0
Long term-stability (−80 °C for 52 days)	101.5 ± 1.9	105.87 ± 2.1

* *n* = 6.

**Table 6:** Stability of ISO in tissue homogenates *.

Homogenate	Concentration (ng/mL)	Stability Remaining (%, Mean ± SD)	
		Short-Term Stability (6 h on Ice)	Post-Preparative Stability (More Than 24 h at 4 °C)
Liver	45.0	102.6 ± 2.1	104.9 ± 2.2
	1600	97.8 ± 1.2	94.9 ± 1.0
Heart	45.0	93.8 ± 3.4	-
	1600	105.7 ± 2.0	-
Kidney	45.0	96.8 ± 2.6	-
	1600	97.4 ± 2.8	-
Brain	45.0	101.3 ± 2.1	-
	1600	95.9 ± 1.7	-
Spleen	45.0	92.7 ± 6.0	-
	1600	94.3 ± 4.3	-
Stomach	45.0	95.0 ± 2.9	-
	1600	97.2 ± 2.2	-
Lung	45.0	97.3 ± 4.0	-
	1600	101.3 ± 1.7	-
Fat	45.0	101.1 ± 4.4	-
	1600	96.8 ± 2.5	-
Small Intestine	45.0	91.8 ± 4.8	-
	1600	92.9 ± 3.8	-
Large Intestine	45.0	92.6 ± 2.9	-
	1600	90.3 ± 3.6	-
Muscle	45.0	101.6 ± 5.5	-
	1600	95.0 ± 2.5	-

* *n* = 6.

## Data Availability

The data presented in this study are available on request from the corresponding authors.

## Supplementary Materials

The following supporting information can be downloaded at: https://www.mdpi.com/article/10.3390/molecules30173635/s1, Figure S1: Representative chromatograms of mouse cardiac homogenate samples; Figure S2: Representative chromatograms of mouse lung homogenate samples; Figure S3: Representative chromatograms of mouse stomach homogenate samples; Figure S4: Representative chromatograms of mouse spleen homogenate samples; Figure S5: Representative chromatograms of mouse kidney homogenate samples; Figure S6: Representative chromatograms of mouse large intestine homogenate samples; Figure S7: Representative chromatograms of mouse fat homogenate samples; Figure S8: Representative chromatograms of mouse small intestine homogenate samples; Figure S9: Representative chromatograms of mouse muscle homogenate samples. Table S1: Accuracy and precision of ISO in tissue homogenates.

## Abbreviations

The following abbreviations are used in this manuscript:

COPD	Chronic obstructive pulmonary disease
HPLC	High performance liquid chromatography
ISO	Isorhapontigenin
LC-MS/MS	Liquid chromatography–tandem mass spectrometry
LLOQ	Lower limit of quantification
QC	Quality control
RES	Resveratrol
RSD	Relative standard deviation
*R*^2^	Correlation coefficient
UV	Ultraviolet
