# Supplementary material for: A Simple and Reliable Method for the Determination of Isorhapontigenin in Murine Biological Matrices: Application in a Tissue Distribution Study
Source: Molecules. 2025 Sep 5;30(17):3635. doi: 10.3390/molecules30173635 (PMC12430278; doi:10.3390/molecules30173635)
Supplement: Supplementary file 1 [file molecules-30-03635-s001.zip › molecules-3756126-supplementary.pdf]

Supplementary Materials

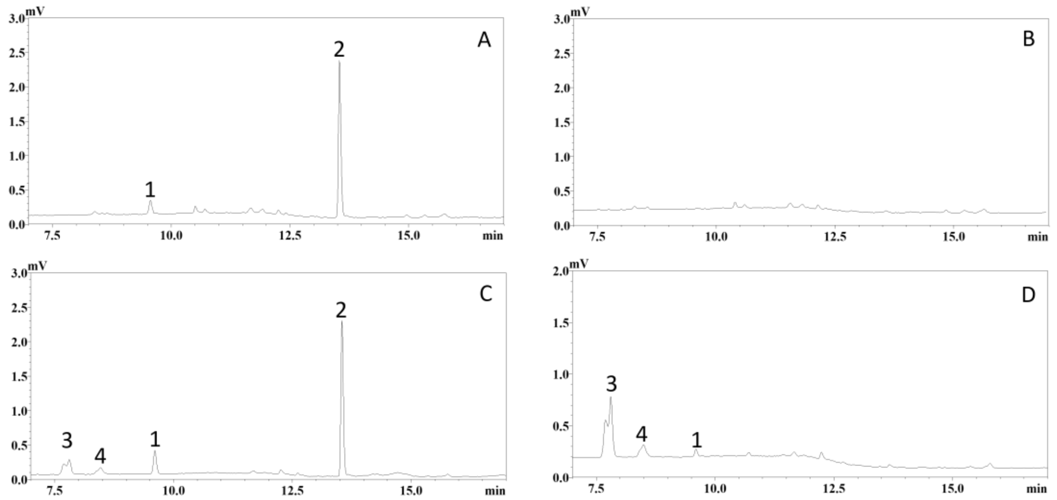

Figure S1. Representative chromatograms of mouse cardiac homogenate samples. Ultraviolet absorbance at 325 nm was recorded for: (A) blank mouse cardiac homogenate spiked with ISO (corresponding to 75 ng/mL in homogenate and 450 ng/mL in tissue) and *trans*-stilbene (internal standard, corresponding to 1200 ng/mL in homogenate); (B) blank mouse cardiac homogenate; (C) cardiac sample collected 20 minutes after oral administration of ISO (200 μmol/kg) with internal standard; and (D) cardiac sample collected 20 minutes after oral administration of ISO (200 μmol/kg) without internal standard. Peaks: 1, ISO; 2, internal standard; 3 and 4, unidentified metabolites.

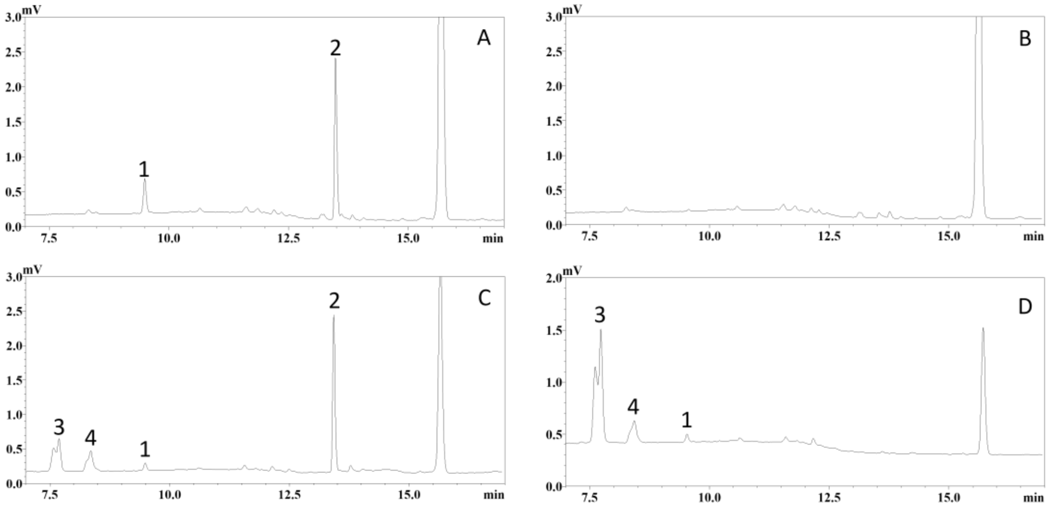

Figure S2. Representative chromatograms of mouse lung homogenate samples. Ultraviolet absorbance at 325 nm was recorded for: (A) blank mouse lung homogenate spiked with ISO (corresponding to 75 ng/mL in homogenate and 450 ng/mL in tissue) and *trans*-stilbene (internal standard, corresponding to 1200 ng/mL in homogenate); (B) blank mouse lung homogenate; (C) lung sample collected 20 minutes after oral administration of ISO (200 μmol/kg) with internal standard; and (D) lung sample collected 20 minutes after oral administration of ISO (200 μmol/kg) without internal standard. Peaks: 1, ISO; 2, internal standard; 3 and 4, unidentified metabolites.

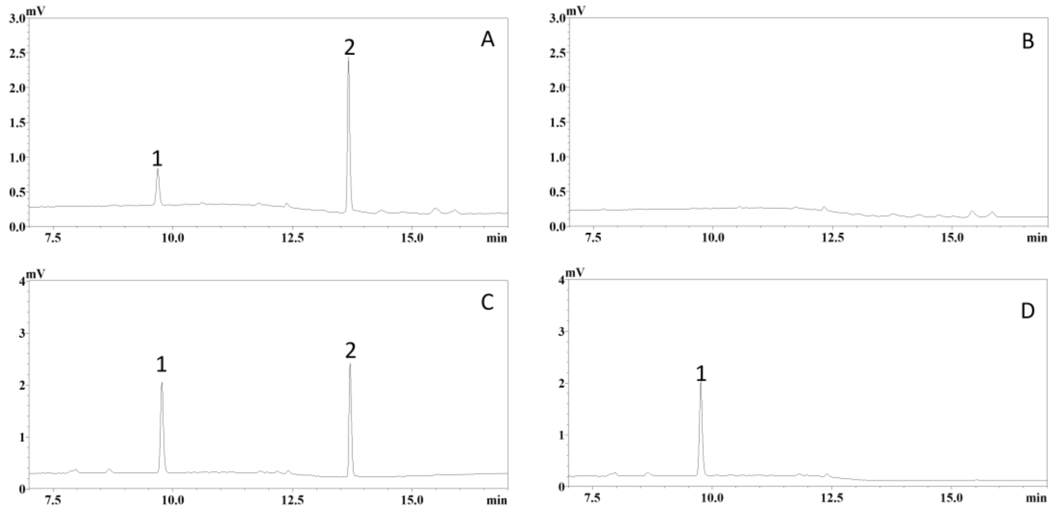

Figure S3. Representative chromatograms of mouse stomach homogenate samples. Ultraviolet absorbance at 325 nm was recorded for: (A) blank mouse stomach homogenate spiked with ISO (corresponding to 75 ng/mL in homogenate and 450 ng/mL in tissue) and *trans*-stilbene (internal standard, corresponding to 1200 ng/mL in homogenate); (B) blank mouse stomach homogenate; (C) stomach sample collected 20 minutes after oral administration of ISO (200 μmol/kg) with internal standard; and (D) stomach sample collected 20 minutes after oral administration of ISO (200 μmol/kg) without internal standard. Peaks: 1, ISO; 2, internal standard; 3 and 4, unidentified metabolites.

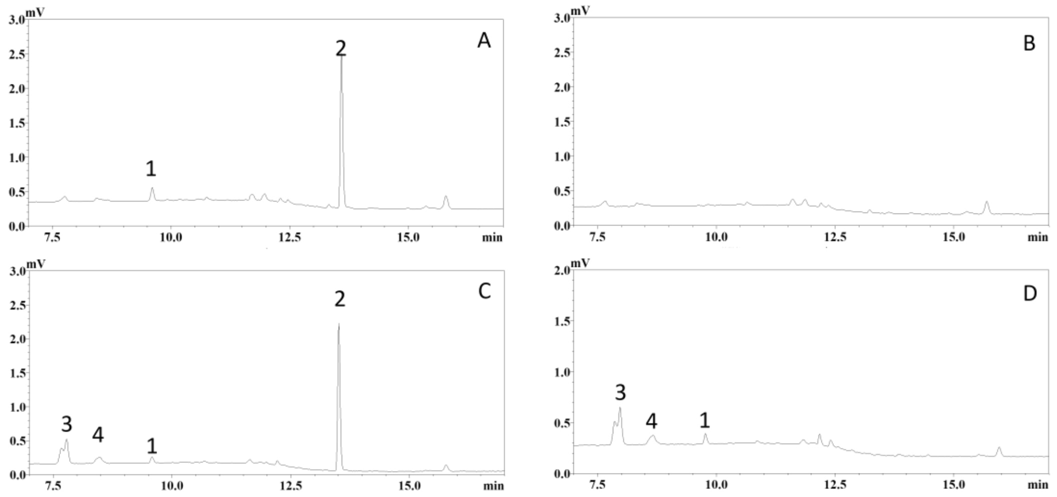

Figure S4. Representative chromatograms of mouse spleen homogenate samples. Ultraviolet absorbance at 325 nm was recorded for: (A) blank mouse spleen homogenate spiked with ISO (corresponding to 75 ng/mL in homogenate and 450 ng/mL in tissue) and *trans*-stilbene (internal standard, corresponding to 1200 ng/mL in homogenate); (B) blank mouse spleen homogenate; (C) spleen sample collected 20 minutes after oral administration of ISO (200 μmol/kg) with internal standard; and (D) spleen sample collected 20 minutes after oral administration of ISO (200 μmol/kg) without internal standard. Peaks: 1, ISO; 2, internal standard; 3 and 4, unidentified metabolites.

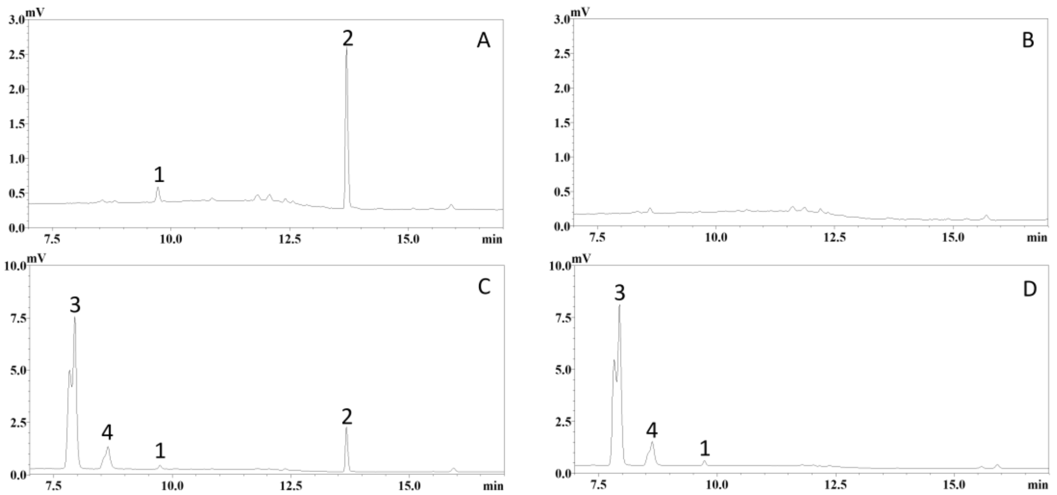

Figure S5. Representative chromatograms of mouse kidney homogenate samples. Ultraviolet absorbance at 325 nm was recorded for: (A) blank kidney homogenate spiked with ISO (corresponding to 75 ng/mL in homogenate and 450 ng/mL in tissue) and *trans*-stilbene (internal standard, corresponding to 1200 ng/mL in homogenate); (B) blank mouse kidney homogenate; (C) kidney sample collected 20 minutes after oral administration of ISO (200 μmol/kg) with internal standard; and (D) kidney sample collected 20 minutes after oral administration of ISO (200 μmol/kg) without internal standard. Peaks: 1, ISO; 2, internal standard; 3 and 4, unidentified metabolites.

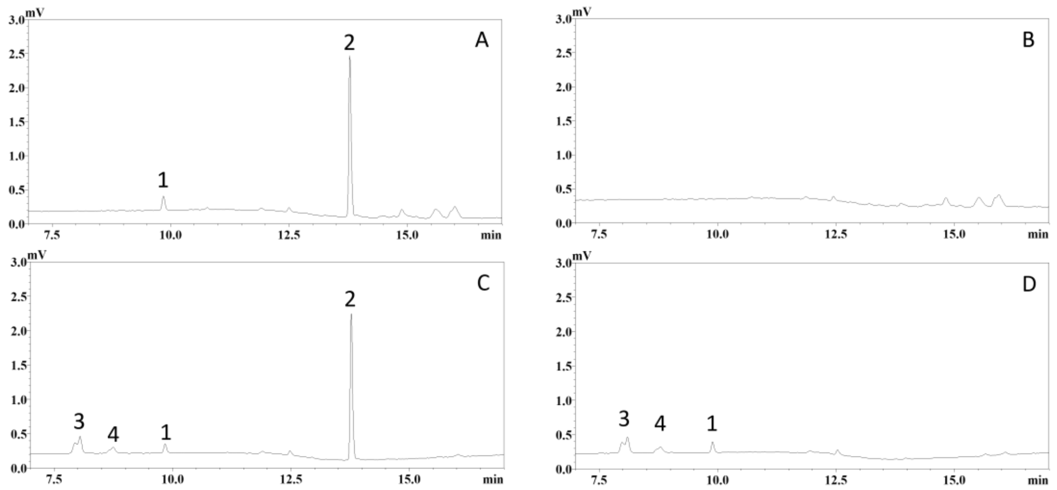

Figure S6. Representative chromatograms of mouse large intestine homogenate samples. Ultraviolet absorbance at 325 nm was recorded for: (A) blank large intestine homogenate spiked with ISO (corresponding to 75 ng/mL in homogenate and 450 ng/mL in tissue) and *trans*-stilbene (internal standard, corresponding to 1200 ng/mL in homogenate); (B) blank mouse large intestine homogenate; (C) large intestine sample collected 20 minutes after oral administration of ISO (200 µmol/kg) with internal standard; and (D) large intestine sample collected 20 minutes after oral administration of ISO (200 µmol/kg) without internal standard. Peaks: 1, ISO; 2, internal standard; 3 and 4, unidentified metabolites.

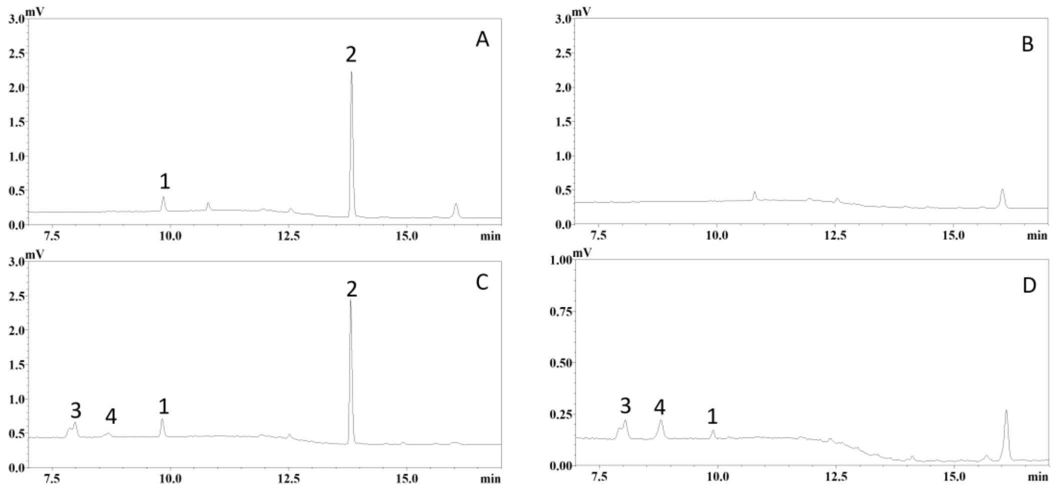

Figure S7. Representative chromatograms of mouse fat homogenate samples. Ultraviolet absorbance at 325 nm was recorded for: (A) blank fat homogenate spiked with ISO (corresponding to 75 ng/mL in homogenate and 450 ng/mL in tissue) and *trans*-stilbene (internal standard, corresponding to 1200 ng/mL in homogenate); (B) blank mouse fat homogenate; (C) fat sample collected 20 minutes after oral administration of ISO (200 μmol/kg) with internal standard; and (D) fat sample collected 20 minutes after oral administration of ISO (200 μmol/kg) without internal standard. Peaks: 1, ISO; 2, internal standard; 3 and 4, unidentified metabolites.

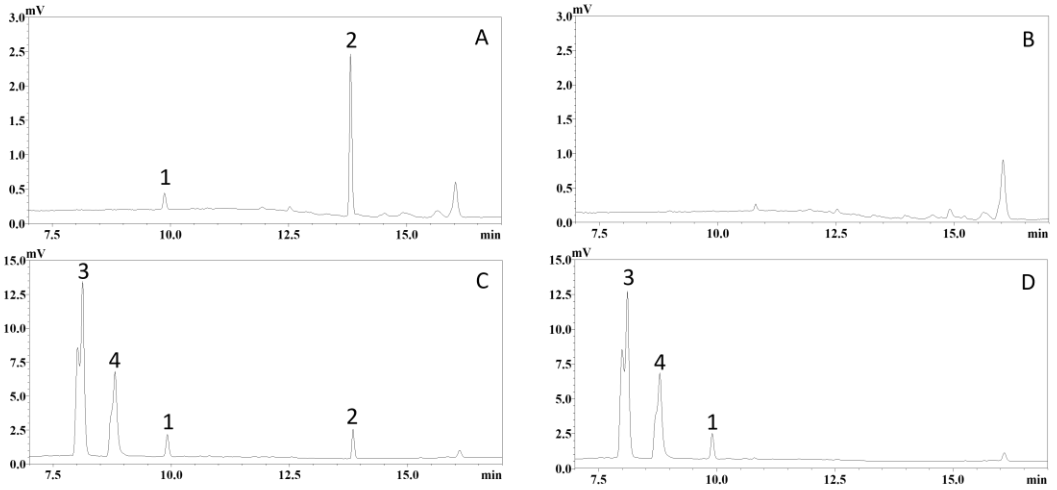

Figure S8. Representative chromatograms of mouse small intestine homogenate samples. Ultraviolet absorbance at 325 nm was recorded for: (A) blank small intestine homogenate spiked with ISO (corresponding to 75 ng/mL in homogenate and 450 ng/mL in tissue) and *trans*-stilbene (internal standard, corresponding to 1200 ng/mL in homogenate); (B) blank mouse small intestine homogenate; (C) small intestine sample collected 20 minutes after oral administration of ISO (200 μmol/kg) with internal standard; and (D) small intestine sample collected 20 minutes after oral administration of ISO (200 μmol/kg) without internal standard. Peaks: 1, ISO; 2, internal standard; 3 and 4, unidentified metabolites.

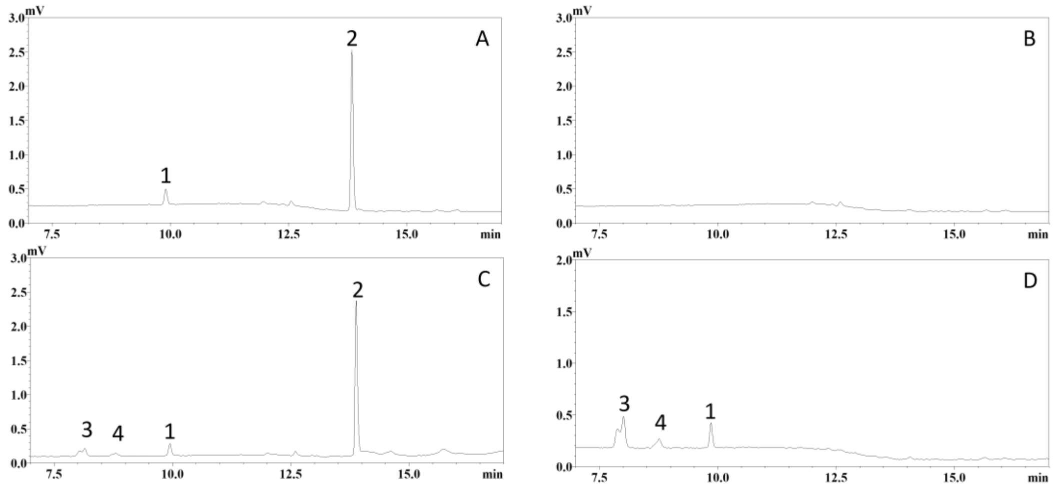

Figure S9. Representative chromatograms of mouse muscle homogenate samples. Ultraviolet absorbance at 325 nm was recorded for: (A) blank muscle homogenate spiked with ISO (corresponding to 75 ng/mL in homogenate and 450 ng/mL in tissue) and *trans*-stilbene (internal standard, corresponding to 1200 ng/mL in homogenate); (B) blank mouse muscle homogenate; (C) muscle sample collected 20 minutes after oral administration of ISO (200 μmol/kg) with internal standard; and (D) muscle sample collected 20 minutes after oral administration of ISO (200 μmol/kg) without internal standard. Peaks: 1, ISO; 2, internal standard; 3 and 4, unidentified metabolites.

Table S1-Accuracy and precision of ISO in tissue homogenates

Matrices	Concentration （ng/mL）	Intra-day(n=6)		
		Observed Concentration (Mean ± SD, ng/mL)	Precision (RSD%)	Mean Accuracy (%)
Heart	15.0	16.5±1.2	7.5	109.9
	45.0	47.2±1.9	4.0	104.9
	175	184.2±2.9	1.6	105.2
	800	817.2±35.2	4.3	102.2
	1600	1746.8±45.0	2.6	109.2
Kidney	15.0	15.4±1.0	6.5	102.9
	45.0	46.6±1.6	3.4	103.5
	175	174.2±5.0	2.9	99.5
	800	843.8±20.4	2.4	105.5
	1600	1555.2±36.3	2.3	97.2
Brain	15.0	16.5±1.6	9.8	109.9
	45.0	48.7±2.3	4.8	108.2
	175	183.7±10.3	5.6	105.0
	800	822.8±51.5	6.3	102.9
	1600	1643.3±34.5	2.1	102.7
Spleen	15.0	13.1±1.3	9.7	87.6
	45.0	42.1±1.8	4.4	93.5
	175	177±3.6	2.0	101.1
	800	797.8±12.2	1.5	99.7
	1600	1648.5±25.7	1.6	103.0
Stomach	15.0	14.6±1.2	8.0	97.6
	45.0	44.7±2.0	4.4	99.4
	175	168.7±6.4	3.8	96.4
	800	798.7±27.1	3.4	99.8
	1600	1561.5±49.9	3.2	97.6
Lung	15.0	16.4±1.2	7.3	109.2
	45.0	47.7±2.8	5.8	106.0
	175	183.3±4.7	2.6	104.8
	800	833.8±10.6	1.3	104.2
	1600	1626±34.4	2.1	101.6
Fat	15.0	15.4±1.0	6.3	102.7
	45.0	48.6±2.1	4.4	107.9
	175	163.7±1.5	0.9	93.5
	800	826.3±16.3	2.0	103.3
	1600	1711.7±71.1	4.2	107.0

Table S1-Continued

Biosample	Concentration（ng/mL）	Intra-day(n=6)		
		Observed Concentration (Mean ± SD, ng/mL)	Precision (RSD%)	Mean Accuracy (%)
Small Intestine	15.0	15.3±1.5	9.9	101.7
	45.0	47.4±2.0	4.1	105.3
	175	182.0±2.3	1.3	104.0
	800	788.2±19.0	2.4	98.5
	1600	1583.0±18.2	1.2	98.9
Large Intestine	15.0	14.6±4.1	27.8	97.4
	45.0	47.2±2.3	4.8	104.9
	175	178.2±1.7	1.0	101.8
	800	771.2±21.9	2.8	96.4
	1600	1547.2±13.1	0.9	96.7
Muscle	15.0	15.6±0.8	5.2	103.8
	45.0	46.1±1.2	2.6	102.5
	175	173.4±15.2	8.8	99.1
	800	766.0±37.8	4.9	95.8
	1600	1428.5±22.5	1.6	89.3
